# Peroxisome Proliferator-Activated Receptor Alpha (PPAR-α) as a Regulator of the Angiogenic Profile of Endometriotic Lesions

**DOI:** 10.7759/cureus.22616

**Published:** 2022-02-25

**Authors:** Vasilios Pergialiotis, Maximos Frountzas, Zacharias Fasoulakis, George Daskalakis, Mairi Chrisochoidi, Konstantinos Kontzoglou, Despoina N Perrea

**Affiliations:** 1 First Department of Obstetrics and Gynecology, National and Kapodistrian University of Athens, Athens, GRC; 2 First Propaedeutic Department of Surgery, Hippocration General Hospital, National and Kapodistrian University of Athens, School of Medicine, Athens, GRC; 3 Department of Obstetrics and Gynecology, National and Kapodistrian University of Athens, Athens, GRC; 4 Laboratory of Experimental Surgery and Surgical Research, National and Kapodistrian University of Athens, Athens, GRC; 5 Second Department of Propedeutic Surgery, National and Kapodistrian University of Athens, Athens, GRC; 6 Laboratory of Experimental Surgery and Surgical Research, National and Kapodistrian University of Athens, School of Medicine, Athens, GRC

**Keywords:** angiogenesis, endometrioma, endometriosis, peroxisome proliferator-activated receptor (ppar), ppar

## Abstract

Endometriosis is a disease that affects a significant proportion of women and its infiltrative pattern is entirely dependent on the vascular supply of lesions. Several factors seem to trigger the process of angiogenesis in endometriotic lesions. During the last years, peroxisome proliferator-activated receptors (PPARs), a group of nuclear proteins that regulate gene transcription and that seem to regulate energy consumption and expenditure, have been also implicated in the pathophysiology of angiogenesis. Their ability to regulate the course of cancer and improve the survival rates of patients has been extensively studied and seems to be partially dependent on alteration of the vascular supply of malignant lesions. Research in the field of endometriosis is scarce in the international literature and mainly focused on PPAR-gamma. However, indirect evidence suggests that PPAR-alpha (PPAR-α) may also regulate the vascular supply of endometriotic lesions as well. Specifically, PPAR-α agonists seem to downregulate angiogenesis by increasing the expression of several anti-angiogenic molecules, including thrombospondin-1 (TSP-1) and gypenoside 140 (gp140), as well as factors that are involved in the mitogen-activated protein kinase cascade. In the present article, we summarize existing indirect and direct evidence that indicates the existence of an association between the expression of PPAR-α and endometriosis to help future research in this field.

## Introduction and background

Endometriosis is a disease that affects a significant proportion of women with a cumulative prevalence that reaches approximately 6.0% by the age of 40-44 years [1,2]. The disease is often debilitating, as in its severest forms, it affects the quality of life of women, with pain being its most prevalent side effect. Several factors seem to affect the pathophysiology of the disease including inflammatory factors, hormones, growth factors, and alteration of genetic and epigenetic pathways [3,4]. Recently, Koninckx et al. described the genetic/epigenetic theory of endometriosis and suggested an interplay between the oxidative stress observed during menstruation and the formation of microscopical lesions that are characterized by a cascade of effects that affect the transcription of genes [5]. As an infiltrative disease that often resembles the pattern of dissemination of cells of various cancer forms, endometriosis is also directly affected by the vascular supply of lesions. Healy et al. were the first to describe the angiogenic process of endometriosis as a pathophysiological requirement that would regulate the course and severity of the disease [6]. Since then, several angiogenic factors have been proposed as diagnostic biomarkers that would help detect endometriosis including vascular endothelial growth factor (VEGF), nerve growth factor (NGF), fibroblast growth factor 2 (FGF-2), leptin, insulin-like growth factor-binding protein 3 (IGFBP-3), glycodelin, macrophage colony-stimulating factor (M-CSF), angiopoietin-1 and angiopoietin-2, microvessel density (MVD), endoglin, and thrombospondin-1 [7,8].

Peroxisome proliferator-activated receptors (PPARs) constitute a group of nuclear proteins that regulate gene transcription [9]. Their function has been described in various diseases and three main isoforms exist: PPAR-alpha (PPAR-α), which is predominantly expressed in the liver; PPAR-gamma (PPAR-γ), which regulates energy storage; and PPAR-delta, which regulates energy expenditure. PPAR-α can be found also in abundance in endothelial cells and immune-type cells, including the monocytes macrophages [10,11]. In the present article, we describe the potential pathophysiological contribution of PPAR-α in the pathophysiology of endometriosis, as we believe that these receptors may regulate the angiogenic profile of endometriotic lesions.

## Review

Search strategy

To obtain information from the international literature, we searched Medline (1966-2021), Scopus (2004-2021), ClinicalTrials.gov (2008-2021), Embase (1980-2021), Cochrane Central Register of Controlled Trials (CENTRAL) (1999-2021), and Google Scholar (2004-2021) databases for relevant articles trying to obtain the latest information in the field.

Current evidence

PPARs have been linked with aging since 1999 as the peroxisomal decline was found to be directly linked with the decline of PPAR-α levels [12-14]. Previous researchers described that the process behind this association may rely on the free radical theory of aging, which results in oxidative stress, insulin resistance, inflammation, and ultimately atherosclerosis [15]. Specifically, PPAR-α seems to limit the expression of inflammatory genes that regulate the production of acute-phase proteins, cytokines, etc. [16].

Endothelial aging may significantly alter angiogenesis and PPARs exhibit an interplay between these two processes [17]. Specifically, activation of the angiogenic profile of endothelial progenitor cells is directly related to the activity of PPAR-β/δ [18]. The process is regulated through the activation of matrix metalloproteinases and other molecules including tetrahydrobiopterin [19,20].

Correlation between PPAR-α expression and angiogenesis

**Figure 1:**
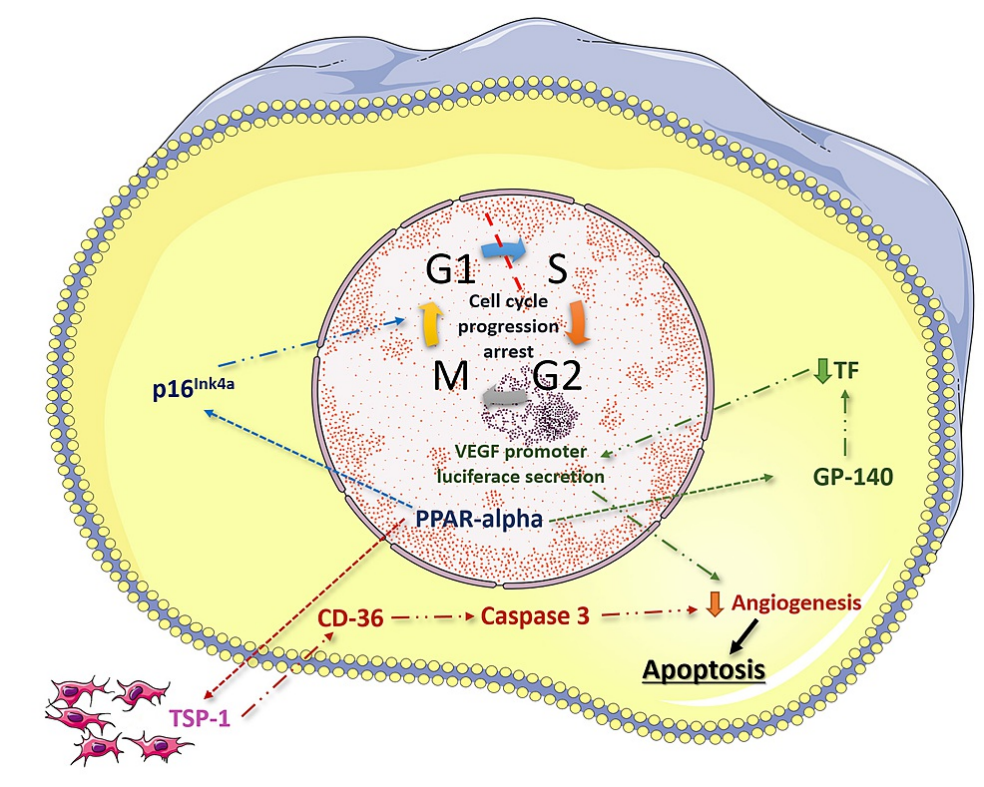
Pathophysiological pathways that correlate PPAR-α with endometriosis. PPAR-α upregulates thrombospondin-1 (TSP-1) expression, which consequently triggers the caspase pathway, therefore, resulting in apoptosis of endothelial cells, reduced angiogenesis, and downregulation of tumor progression. Upregulation of gypenoside 140 (GP-140) results in reduced promoter luciferase secretion of the VEGF gene, which also reduces the process of angiogenesis. Activation of the P16Ink4A pathway results in cell cycle arrest and reduced endothelial proliferation. PPAR-α, peroxisome proliferator-activated receptor alpha; VEGF, vascular endothelial growth factor; TF, tissue factor.

PPAR-α increases the expression of several anti-angiogenic molecules, including thrombospondin-1 (TSP-1) and gypenoside 140 (gp-140), as well as several factors that are involved in the mitogen-activated protein kinase cascade (Figure 1) [21]. Thrombospondins function as regulators of angiogenesis and seem to act as matricellular proteins as they interact with cell-surface receptors and several other molecules including proteases and growth factors. Ultimately, this process results in the apoptosis of endothelial cells through activation of CD36 [22]. Similarly, gp-140 results in the decrease of tissue factor (TF) that consequently downregulates the expression of the VEGF and TSP-1 genes, which help maintain the vascular supply of tumors [23]. Another pathophysiological pathway that correlates PPAR-α with the downregulation of angiogenesis is its inhibitory effect on vascular smooth muscle proliferation [24]. The process is triggered through the activation of p16(INK4a) a protein that acts as a cyclin-dependent kinase inhibitor that arrests cell cycle progression at the G1/S phase.

The contribution of PPAR-α in cancer development and progression has been reported in several studies. Specifically, ligands of this receptor seem to inhibit the growth of various cancer forms, including bladder, testicular, colorectal, and prostate cancer [25-28]. Accumulated evidence supports that PPAR-α ligands may have an antitumoral capacity, which is directly related to their anti-inflammatory and anti-angiogenic properties. This is mainly supported by studies that prove that PPAR-α agonists have the ability to inhibit VEGF signaling through repression of Sp1-site-dependent DNA binding and transactivation as well as through metabolism of epoxyeicosatrienoic acids (EETs) to pro-angiogenic lipids [29-31]. This theory has been investigated in both in vivo andin vitro models following the administration of fibrates that inhibit the angiogenic process [32].

Endometrial expression of PPAR-α has been observed in cancer models in vivo. Specifically, it seems that their expression in the normal endometrium is modes whereas, this gradually increases during the transition to atypical hyperplasia and cancer [33]. This effect seems to be triggered by an increase in angiogenesis that is manifested by an increased expression of PPAR-α in vascular endothelial cells as well as endometrial glandular and tumor cells. To date, it remains unknown if the expression of the PPAR-α protein is the cornerstone of this regulatory mechanism or if this relies on the abundance of PPAR-α receptors. However, it seems that even in the in vitro models with down-regulation of the receptor, the abundance of PPAR-α protein may compensate adequately, therefore, maintaining the angiogenic potency that is desired to enhance tumorigenesis [33].

In the field of ovarian cancer, activation of PPAR-α has been involved with suppression of the hypoxia-inducible factor 1 alpha (HIF-1a), a process that was confirmed following the use of proteasome inhibitors, which reversed this process [34]. The latter protein (HIF-1a) belongs in a family of transcription factors, which are aggregated following the detection of decreased oxygen availability in the cellular environment. Induction of hypoxia is believed to preserve the potency of stem cells for a long period of time, which have low metabolic needs [35]. Quiescent endometrial progenitor cells have been previously implicated in theories concerning the development of endometriosis; however, experimental studies have not proven this theory yet [36,37].

Evidence in PPAR-α-deficient transgenic models

In 2007, Kaipainen et al. observed that PPAR-α-deficient mice exhibit diminished tumor development, a process that may be regulated by CYP2C9 epoxygenase expression [38]. The process is supposed to alter tubulogenesis in endothelial cells, which are essential during neo-vascularization.

Evidence from clinical studies

Both fenofibrate and bezafibrate have been used in clinical studies and cancer cell lines and evidence suggests that these PPAR-α ligands may benefit survival rates of cancer patients [39,40]. In the field of ovarian cancer, modulation of cholesterol homeostasis seems to modulate platinum sensitivity [41], and in 2013, Fang et al. supported that this process may be triggered by the interplay between apolipoprotein A-I (ApoA-I)-binding protein (AIBP) and angiogenesis [42]. In a large cohort study that was based on survival outcomes of 2,195 women with ovarian cancer, researchers observed that statin use was associated with a lower risk of death (OR: 0.74%, 95% CI: 0.61-0.91%) [43]. Another observational study that was based on the Surveillance, Epidemiology, and End Results (SEER) registries and which recruited 1,431 ovarian cancer patients also suggested that women under lipophilic statin use had significant improvement in their overall survival rates (hazard ratio: 0.66, 95% CI: 0.55-0.81) [44].

PPARs in endometriosis

In the field of endometriosis, evidence that correlates PPARs with the pathophysiology of the disease is limited to a handful of studies. The majority of research is focused on PPAR-γ and evidence suggests that this receptor leads to repression of VEGF expression in human endometrial cells. Aside from that, PPARs seem to reduce tumor necrosis factor-alpha (TNF-a)-induced interleukin 8 (IL-8) production in endometriotic stromal cells [45]. The later protein seems to be elevated in the peritoneal tissue of women with endometriosis and seems to act both indirectly as a chemoattractant that stimulates leucocytes to express growth facts as directly in endometriotic cells [46].

Experimental research is extremely limited and mainly covers the effect of PPAR-γ on the course of endometriosis. Peeters et al. observed that endometrial cells express the PPAR-γ protein and following the performance of an in vitro study, they found that rosiglitazone inhibited VEGF expression by approximately 20-50% [47]. Almost synchronous to that study was the one conducted by Demirturk et al. in an experimental rat model [48]. The authors observed that rosiglitazone negatively influenced the induction of endometriosis as both post-treatment spherical volumes of lesions (64.00 mm^3^ (interquartile range (IQR): 354.42) vs. 41.60 mm^3^ (IQR: 37.87), p = 0.018) as well as explant weights were significantly smaller among animals treated with the PPAR-γ agonist. In primates, Lebovic et al. observed in 2007 that rosiglitazone effectively reduced the size of the surface area of peritoneal lesions [49].

The impact of polymorphisms remains poorly explored; however, there seems to be evidence that supports certain alleles (such as 161CC and 161C of the Pro12Ala locus in exon B of the PPAR-γ gene) that might influence the course and severity of endometriosis [50].

In the clinical setting, only one case report has been published to date in which an abdominal wall scar extrapelvic endometriosis was studied [51]. The authors reported that following pathology analysis of both the eutopic and ectopic endometrium, aberrant expression was observed, which indirectly supports the hypothesis of the potential effect of PPAR agonists in these patients.

## Conclusions

To date, the impact of PPAR-α in the pathophysiology of endometriosis remains unexplored. Current evidence indirectly links its activity with the process of angiogenesis in various diseases, including ovarian cancer, which resembles endometriosis in terms of disease progression, and scientific interest in this field gains ground. In the clinical setting, PPAR-α agonists have proven their impact on ovarian cancer survival. Taken together with their potent anti-angiogenic activity, they seem to be of special interest in the field of endometriosis as well. Further research is warranted in a pre-clinical setting to evaluate whether these drugs can help minimize the burden of disease and to evaluate their actual impact on the angiogenic profile of endometriotic implants. While in vitro studies have clearly depicted this, we believe that further in vivo studies are needed in animal models, both transgenic (lack of PPAR-α expression) as well as following administration of PPAR-α agonists (including clofibrate and bezafibrate, which have proven their anti-angiogenic activity in other diseases).
